# Aspirations for teaching career: what we need to know and what we need to do

**DOI:** 10.3389/fpsyg.2025.1525153

**Published:** 2025-03-26

**Authors:** Ana Daniela Silva, Maria do Céu Taveira

**Affiliations:** School of Psychology, University of Minho, Braga, Portugal

**Keywords:** career aspirations, teaching career, vocational psychology, career choice, career exploration

## Introduction

The teaching profession is fundamental to the functioning of society and to guaranteeing quality education, as set out in Sustainable Developmental Goal (SDG) 4 of the 2030 Agenda. However, the scientific literature in the field and various government reports refer to a global teacher shortage crisis (Arnold and Rahimi, 2025; Flores and Craig, 2023; Monteiro et al., 2020). Although the phenomenon is not new, data from some countries show the urgency of acting on the problem due to its worsening and the consequences for education systems and society in the short and medium term (Flores and Craig, 2023; Mockler, 2024; Rahimi and Arnold, 2024; Rodrigues et al., 2019). Between 2015 and 2022, the share of students whose principals reported shortages rose from 29 to 46.7% across the OECD (OECD, 2024). According to UNESCO (2017), by 2030, it is estimated that the world will need an additional 69 million teachers to provide inclusive and equitable quality education for all. In addition to the aging of the teaching population, the low recognition of the profession by society and the low attractiveness of this career option for younger people are also factors in the problem (Dotta et al., 2025; Flores and Craig, 2023).

The vocational literature refers to the importance of aspirations for career choices and how personal and contextual factors can influence these throughout the life cycle (Brown and Lent, 2021). In this opinion article, we will present some aspects that characterize the teaching profession and its challenges and reflect on the role of career aspirations in developing professional interests and choices that could favor the recruitment and retention of these much-needed professionals.

We believe that this perspective on the issue, based on Vocational Psychology and Career Development, can contribute to what we need to know and what can be done to address this important social problem. In this way, the aim is to contribute to developing research lines in Vocational Psychology that address the mentioned challenges.

### The teaching career

The right to education may be considered the most fundamental for a human life. Although there seems to be a consensus on the importance of education for societies, two terms are commonly used concerning the status of the teaching profession worldwide: decline and (lack of) recognition (Flores and Craig, 2023).

In fact, both the early stage of a teaching career and the growing shortage of professionals have been the subject of analysis in various regions of the world, as well as in many member countries of the Organization for Economic Co-operation and Development (OECD; Watt and Richardson, 2008). The complexity of this issue is also expressed through concerns related to the quality of professionals, the aging of the teaching population, and negative representations of the profession in the media. All of these factors determine the career path of professionals in education (OECD, 2005).

For example, a national study in Portugal, a southwest European country, highlights the dissatisfaction and perceived lack of recognition of the teaching profession (Rodrigues et al., 2019). Such results highlight the emergence of precarious situations, coexisting with other social, institutional, and personal factors affecting these professionals: working conditions, professional and personal satisfaction, motivation, stress, and insecurity (Flores and Craig, 2023; Lopes and Oliveira, 2022; Mota et al., 2021; Nunes et al., 2021; Reis et al., 2023; Rodrigues et al., 2019; Slemp et al., 2020). Noteworthy is that the competition for accessing the career is complex. Carried out centrally, with the residual possibility of choice, creating mobility with possible harm to teachers and schools' work, these lead to late placements, dislocation from the teachers' residence, and lack of teachers in some regions (Rodrigues et al., 2019). Moreover, the teaching career structure integrates 10 levels with differentiated remuneration indexes. Career progression/salary increases are the most common incentives in most European education systems. However, the distribution of Portuguese teachers by level reveals that 58.4% are in the first four levels (Nunes et al., 2021; Rodrigues et al., 2019).

This scenario, marked by growing tensions and the ongoing struggle to have their rights recognized as professionals over the years, along with the ongoing efforts to gain recognition for the profession in society, has become a constant theme in our daily lives. This could influence how young people perceive the profession.

This is evident in the decrease of graduates in teacher training courses and the intentions of students in choosing this profession (Reis et al., 2023). In the same country, data from 2019 indicate that students entering courses in the area of Education have one of the lowest averages in the Portuguese national exams (Rodrigues et al., 2019). This aligns with the TALIS 2018 data suggesting that teaching was a first career choice for 66% of survey respondents across the OECD, but only 26% felt that the profession was valued in society (OECD, 2020). The unattractiveness of the teaching profession for young people aligns with Portuguese parents' professional aspirations for their children. A study showed that around half of Portuguese parents discouraged their children from pursuing the profession, the highest figure among the European Union countries analyzed (Dolton et al., 2018). Even so, according to an OECD (2018) survey, more than 90% of Portuguese teachers named the possibility of influencing the development of students, providing a service to society, and raising the ambitions of the less privileged as the most important factors for choosing their profession.

Portugal's teacher shortage crisis is a significant but not unique example. Compared to other OECD countries, it shares many challenges, particularly in attracting and retaining teachers. However, in the OECD report (2024), compared to other countries, Portugal is barely mentioned in policies aimed at developing campaigns to improve teaching status, which could be important in promoting aspirations for teaching careers. Compared to non-OECD countries, Portugal's challenges are less severe in scale, particularly regarding teacher shortages. Countries in Sub-Saharan Africa and South Asia face much more acute shortages, particularly in primary education, due to lower teacher training and resources (OECD, 2024).

These data justify the focus on the study of teaching careers (Oliveira et al., 2023). However, it is important to continue this research to understand how we can ensure the representativity and quality of this profession, which has a decisive impact on students' performance, professional success, and the country's socioeconomic growth and development (Hanushek and Woessmann, 2020).

### The importance of career aspirations

The vocational literature (Brown and Lent, 2021) has demonstrated the importance of career aspirations in the choices and process of career development. Developing career-relevant interests and aspirations are prominent developmental tasks of the elementary and middle school years, continually revisited and refined in high school and beyond (Lent et al., 1999). Career or occupational aspirations have been conceptualized as a major career developmental task when seeking careers compatible with our self-concepts, i.e., with an individual's perception of their abilities, interests, values, and roles in the world (Patton and Creed, 2007). As proposed in Super's vocational development theory (1990), vocational self-concept plays an important role in choosing careers that match individuals' self-image. The interaction between the person and the environment forms it. In this regard, the social cognitive career theory highlights the role of environmental factors such as opportunities, resources, barriers, financial resources, parental behavior, and school influence on an individual's career interests and choices. Direct and indirect learning experiences, by observing family and community members or how the media portrays a profession, are presented as determining factors (Lent, 2021; Silva et al., 2021). Throughout childhood and adolescence, people increasingly experience varied performance tasks and direct and vicarious exposure, by observing others' experiences, to a widening range of career possibilities, leading to differentiated beliefs regarding one's capabilities in diverse activity domains and an expanded sense of working conditions (Brown and Lent, 2021). The knowledge of professions can also influence the interests and aspirations, enhancing the individual's self-concept development. The self-concept was also associated with future career projection (Holcomb-McCoy and Young, 2012). It was linked to an individual's expression of career-related goals/choices, and early aspiration can be applied to predict later aspiration and, ultimately, the occupational choice people make (Rojewski, 2005). Adolescents' aspirations and expectations predicted adult educational attainment 8 years later (Beal and Crockett, 2010). It is important, therefore, to understand the role of these dimensions in students' (dis)interest in their studies and the teaching profession and to draw practical implications.

## Discussion

The United Nations acknowledges that quality education for all is fundamental to creating a peaceful and prosperous world—providing people with the knowledge and skills they need to stay healthy, get jobs, and foster tolerance. In fact, UNESCO recognizes “education as a public good, a global common good, a fundamental human right and a basis for guaranteeing the realization of other rights” (UNESCO, 2017, p. 19).

Although the problem of teacher shortages and low career aspirations in the teaching profession is well documented in the literature, further studies are still needed that focus on developing career aspirations in teaching throughout students' schooling and academic journey. This knowledge will make it possible to develop career exploration resources that avoid the circumscription of this professional interest and favor the choice of training in this area, as well as academic and professional paths based on involvement and quality.

For example, producing career exploration resources, using career narratives and testimonials adapted for each public age, and encouraging them to view teaching as a desirable and achievable career path, could be an important career education intervention.

However, it is important, in these works, to take into account career development theories that explain the role of career aspirations in the development of career interests and choices (Brown and Lent, 2021), going beyond the efforts of existing literature that focus too much on the effects of social and educational policies to explain this problem (e. g., Flores and Craig, 2023; OECD, 2024). These works should develop efforts to understand and act on the (un)attractiveness of the teaching career, analyzing the phenomenon in an interdisciplinary way, understanding the aspirations for the teaching career in different generations of young people and the factors that influence them, and developing resources for exploring the teaching career that favors committed choices for this training path.

In conclusion, in this scope, Vocational Psychology can offer important contributions to “what we need to know and what we need to do” through studies that promote (i) the empirical evidence of the development of teaching career aspirations over time and its potential impact on teaching profession choice, (ii) the knowledge about students' teaching career aspirations, according to different personal and contextual circumstances; (iii) the development and evaluation of interventions for the promotion of career exploration of the teaching profession.
